# Dr. Knapp’s Treatise

**Published:** 1855-09

**Authors:** 


### DR. KNAPP'S TREATISE.

We have concluded in this number the interesting essay of Dr. Knapp upon a subject of much interest to the profession generally, but particularly to the western practitioner, where the disease is more frequently met with. We are much gratified that it has been so ably considered by the learned author, and in a particular manner the compilation of the literature of the disease. Whether we differ with him or not in the existence of the pathological points which would entitle it to the nosological rank which he assigns it, he deserves infinite credit for the industry with which he has pursued the subject, and the ability which he has exhibited.

Since the publication of the article to which he alludes in his treatise, embracing the facts in relation to that disease which had fallen under our observation up to that time; we have enjoyed the opportunity of a more extended observation of its phenomina, and this additional experience would not justify us in closing in wholly with the author's pathological views of it. This experience, and these views, we will take occasion hereafter to present.

We find upon conversing with our medical brethren on the subject in different localities of the State, that they have met with cases of this disease, all whom are anxious for more light upon a subject heretofore so dark and obscure. To these, the article of Dr. Knapp will prove highly interesting and instructive. The article referred to by Dr. Knapp, was the first recorded notice of the disease in this State, and perhaps, of this region of the west, therefore the report we then made was the result of our own observations wholly and without the aid of any other observer. This too, was the gathering of but two years of experience, not having seen the disease in the more eastern States, which had previously been the field of our labors. Since then, we have added four years more of observation to our previous experience, a part of which was made under painful circumstances.

In our investigations we shall endeavor to present such facts as will bear upon the pathology of this peculiar affection, and those who have promised us the benefit of *their* experience will promptly furnish them. Others, who have met with cases of this nature will please contribute to the means of a still more satisfactory elucidation of the subject. We hope the author will pursue the subject as he has intimated, in order that the profession may be able to fix upon its true pathology in order to its place in the nosological arrangement of diseases.

If it be not dependent upon the puerperal condition, and this fact can be established, it will change entirely the position and the views of the profession heretofore on the subject.

